# Deregulated microRNAs in triple-negative breast cancer revealed by deep sequencing

**DOI:** 10.1186/s12943-015-0301-9

**Published:** 2015-02-10

**Authors:** Yao-Yin Chang, Wen-Hung Kuo, Jui-Hui Hung, Chien-Yueh Lee, Yung-Hua Lee, Ya-Chu Chang, Wen-Chun Lin, Cheng-Ying Shen, Chiun-Sheng Huang, Fon-Jou Hsieh, Liang-Chuan Lai, Mong-Hsun Tsai, King-Jen Chang, Eric Y Chuang

**Affiliations:** Department of Electrical Engineering, Graduate Institute of Biomedical Electronics and Bioinformatics, National Taiwan University, Taipei, Taiwan; Department of Surgery, College of Medicine, National Taiwan University, Taipei, Taiwan; Bioinformatics and Biostatistics Core, NTU Center of Genomic Medicine, Taipei, Taiwan; Institute of Biotechnology, College of Bio-resources and Agriculture, National Taiwan University, Taipei, Taiwan; Department of Obstetrics and Gynecology, College of Medicine, National Taiwan University, Taipei, Taiwan; Department of Physiology, College of Medicine, National Taiwan University, Taipei, Taiwan; Department of Surgery, Cheng Ching General Hospital, Taichung, Taiwan

**Keywords:** Triple-negative breast cancer, Deep sequencing, MicroRNA expression, MicroRNA cluster, miR-130b-5p, *CCNG2*

## Abstract

**Background:**

MicroRNAs (miRNAs) are short, non-coding RNA molecules that play critical roles in human malignancy. However, the regulatory characteristics of miRNAs in triple-negative breast cancer, a phenotype of breast cancer that does not express the genes for estrogen receptor, progesterone receptor, and human epidermal growth factor receptor 2, are still poorly understood.

**Methods:**

In this study, miRNA expression profiles of 24 triple-negative breast cancers and 14 adjacent normal tissues were analyzed using deep sequencing technology. Expression levels of miRNA reads were normalized with the quantile-quantile scaling method. Deregulated miRNAs in triple-negative breast cancer were identified from the sequencing data using the Student’s t-test. Quantitative reverse transcription PCR validations were carried out to examine miRNA expression levels. Potential target candidates of a miRNA were predicted using published target prediction algorithms. Luciferase reporter assay experiments were performed to verify a putative miRNA-target relationship. Validated molecular targets of the deregulated miRNAs were retrieved from curated databases and their associations with cancer progression were discussed.

**Results:**

A novel 25-miRNA expression signature was found to effectively distinguish triple-negative breast cancers from surrounding normal tissues in a hierarchical clustering analysis. We documented the evidence of seven polycistronic miRNA clusters preferentially harboring deregulated miRNAs in triple-negative breast cancer. Two of these miRNA clusters (miR-143-145 at 5q32 and miR-497-195 at 17p13.1) were markedly down-regulated in triple-negative breast cancer, while the other five miRNA clusters (miR-17-92 at 13q31.3, miR-183-182 at 7q32.2, miR-200-429 at 1p36.33, miR-301b-130b at 22q11.21, and miR-532-502 at Xp11.23) were up-regulated in triple-negative breast cancer. Moreover, miR-130b-5p from the miR-301b-130b cluster was shown to directly repress the cyclin G2 (*CCNG2*) gene, a crucial cell cycle regulator, in triple-negative breast cancer cells. Luciferase reporter assays showed that miR-130b-5p-mediated repression of *CCNG2* was dependent on the sequence of the 3′-untranslated region. The findings described in this study implicate a miR-130b-5p-*CCNG2* axis that may be involved in the malignant progression of triple-negative breast cancer.

**Conclusions:**

Our work delivers a clear picture of the global miRNA regulatory characteristics in triple-negative breast cancer and extends the current knowledge of microRNA regulatory network.

**Electronic supplementary material:**

The online version of this article (doi:10.1186/s12943-015-0301-9) contains supplementary material, which is available to authorized users.

## Background

Breast cancer is a heterogeneous disease that can be classified into several histological forms in current clinical practice. The molecular etiologies among different types of breast cancers are largely different, making the treatment of breast cancer difficult. Triple-negative breast cancer, which is characterized by the lack of expression of the estrogen receptor (ER), the progesterone receptor (PR), and the human epidermal growth factor receptor 2 (HER2), is a type of breast cancer with aggressive tumor behavior. Many targeted treatments, including endocrine therapies and HER2-targeted medicine, are not efficacious for triple-negative breast cancer [1,2]. Although previous studies have shown that the vast majority of triple-negative breast cancers display basal-like gene expression features [3,4] the molecular mechanisms driving tumor progression of triple-negative breast cancer still remain unknown.

MicroRNAs (miRNAs) are short (~21mer) non-coding RNA molecules that are important in gene expression regulation [5,6]. The primary role of miRNAs appears to be in the negative regulation of the expression of messenger RNA (mRNA) transcripts. The functional strand of a mature miRNA guides the RNA-induced silencing complex to bind a target mRNA in the 3′-untranslated region (3′-UTR), initiating translational repression, target mRNA cleavage, or mRNA deadenylation of the target gene. Emerging evidence has shown that aberrant miRNA expression plays a critical role in the tumorigenesis of many human cancers [7-9]. Some miRNAs are shown to possess oncogenic characteristics that promote malignancy of human cancers [10], while some have tumor-suppressing abilities to reduce the production of oncogenic proteins [11,12].

The advent of deep sequencing technology allowed us to explore the largely unknown territory of the miRNA transcriptome in triple-negative breast cancer. Sequencing reads of miRNA expression data from 24 triple-negative breast cancers and 14 adjacent normal tissues were analyzed for the presence of deregulated miRNAs in this study. Differentially expressed miRNAs in triple-negative breast cancer were determined by statistical analyses of the sequencing data and were validated using the quantitative reverse transcription PCR (RT-PCR) method. We identified seven polycistronic miRNA clusters in the human genome harboring 29 deregulated miRNAs in triple-negative breast cancer. Furthermore, our work extends the potential target network of miRNAs by showing that the cyclin G2 gene (*CCNG2*) is a direct target of miR-130b-5p from the miR-301b-130b cluster. Forced expression of miR-130b-5p was found to significantly repress the endogenous expression levels of *CCNG2* in triple-negative breast cancer cells. The findings described in this work may provide insights into the miRNA regulatory mechanisms underlying the tumorigenicity of triple-negative breast cancer.

## Results

### A 25-miRNA signature discriminating triple-negative breast cancer from adjacent normal tissue

**Figure 1:**
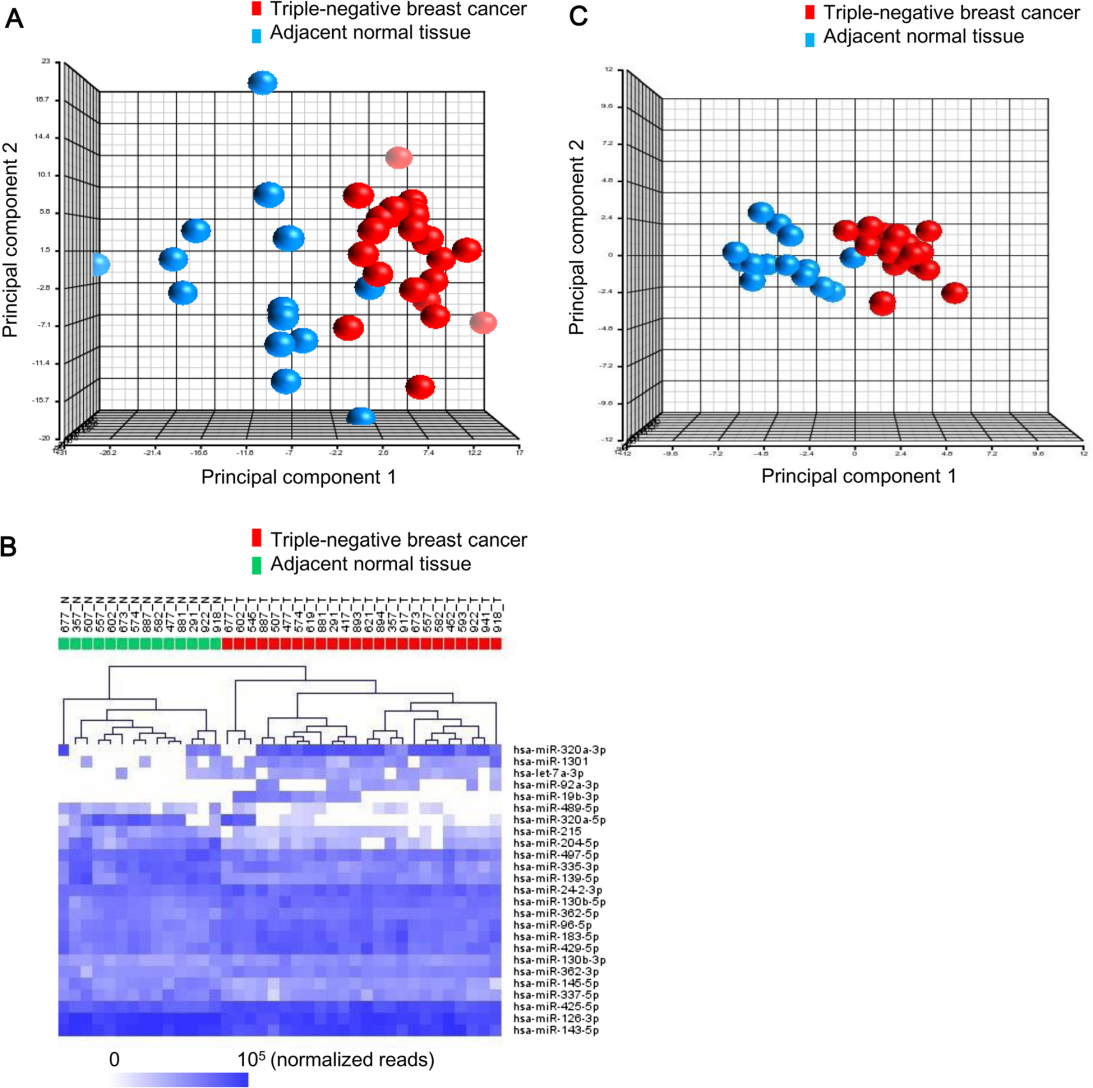
**A 25-miRNA expression signature discriminating between triple-negative breast cancers and adjacent normal tissues.** **(A)** The global miRNA expression portrait from each sample was investigated using principal component analysis. Each data point in the principal component plot is composed of gene expression data of 707 mature miRNAs from a sample. Triple-negative breast cancers are shown in red and adjacent normal tissues are shown in blue. **(B)** Hierarchical clustering analysis of triple-negative breast cancers (red) and adjacent normal tissues (green) was performed using the 25 differentially expressed miRNAs. Each row represents the normalized gene expression data of a miRNA and each column represents a tissue sample. The dendrogram depicts similarities in the gene expression profiles among the samples. **(C)** Triple-negative breast cancers (red) and adjacent normal tissues (blue) are clearly separated into two groups in the principal component analysis using the gene expression data of the 25 differentially expressed miRNAs.

Clinical information on the patients was recorded as shown in Table 1. A total of 113,412,568 miRNA reads were identified from 24 triple-negative breast cancers and 14 adjacent normal tissues after sequence alignment with the human miRNA reference, yielding a median of 2,670,242 miRNA reads per sample from our data (range: 393,305-7,906,634; Additional file 1). Setting a threshold to filter out the miRNAs with extremely low reads (mean expression <5 reads across 38 samples), we identified 707 mature miRNAs. The reads from those 707 miRNAs constituted 99.97% of all miRNA reads in our data for further analysis. The miRNA reads in each sample were normalized using the quantile-quantile scaling method on a log_10_-scale. Normalized expression data from each of the 707 miRNAs was used as input to generate a PCA plot to visually assess the intrinsic variation in the global miRNA profiles among the samples. The PCA plot showed that the global miRNA portrait was able to roughly separate the majority of triple-negative breast cancers from the adjacent normal tissues (Figure 1A).

**Table 1:** **Clinical information on 24 triple-negative breast cancer patients**

**Sample ID**	**Age (years)**	**Stage**	**Grade** ^**a**^	**Tumor size** ^**b**^	**LYM** ^**c**^	**Recurrence status** ^**d**^	**Recurrence-free time (years)**
452_T	54	I	2	1	0	0	6.6
477_T	27	I	3	1	0	0	6.5
593_T	55	I	3	1	0	0	5.9
602_T	44	I	3	1	0	0	5.9
621_T	62	I	2	1	0	0	5.8
894_T	54	I	2	1	0	0	4.6
922_T	63	I	3	1	0	0	4.5
417_T	33	IIA	3	2	0	0	6.8
507_T	54	IIA	3	1	1	0	6.4
545_T	55	IIA	3	2	0	0	6.2
557_T	60	IIA	3	2	0	0	6.1
574_T	57	IIA	2	2	0	0	6.0
582_T	83	IIA	3	2	0	1	3.5
619_T	37	IIA	3	2	0	1	1.8
673_T	61	IIA	2	2	0	1	0.7
887_T	49	IIA	3	2	0	0	4.6
917_T	51	IIA	3	2	0	0	4.5
918_T	38	IIA	3	2	0	0	4.5
941_T	52	IIA	3	1	1	0	4.4
677_T	65	IIB	3	2	1	0	5.5
881_T	58	IIB	3	2	1	0	4.7
893_T	55	IIB	3	2	1	1	1.5
291_T	85	IIIA	3	3	1	1	1.2
357_T	57	IIIC	3	2	1	0	7.1

^a^Grade: 1/2/3, low/intermediate/high.

^b^Tumor size: 1/2/3, <2 cm/2 cm-5 cm/>5 cm.

^c^LYM, lymph node metastasis: 0/1, negative/positive.

^d^Recurrence: 0/1, negative/positive.

Differentially expressed miRNAs between the triple-negative breast cancer group (N = 24) and the adjacent normal tissue group (N = 14) were identified using Student’s t-test and the Holm step down procedure for *p*-value adjustment. A list of the top 25 significantly differentially expressed miRNAs (adjusted *p* <0.05) was determined as shown in Table 2. Average-linkage hierarchical clustering analysis was performed on the samples using the expression data from the 25 miRNAs (Figure 1B). All triple-negative breast cancers and adjacent normal tissues were distinctively divided into two clusters without any misclassification. To examine whether the 25-miRNA signature contained relevant information in classification of triple-negative breast cancers and normal breast tissues in PCA, each sample was projected onto the principal components using the expression data from the 25 miRNAs (Figure 1C). The PCA plot revealed that the triple-negative breast cancers and the adjacent normal tissues were clearly separated into two groups using the 25-miRNA signature.

**Table 2:** **Twenty-five differentially expressed miRNAs between triple-negative breast cancers and adjacent normal tissues** ^**a**^

**miRNA**	**Raw** ***p *** **value**	**Adjusted** ***p*** ** value**	**Triple-negative breast cancer**			**Adjacent normal tissue**		
			**Mean**	**Range**		**Mean**	**Range**	
			**Normalized**	**Min**	**Max**	**Normalized**	**Min**	**Max**
hsa-miR-215	3.21E-08	4.66E-06	32	0	86	321	80	928
hsa-miR-497-5p	2.43E-06	3.49E-04	1980	354	6215	6520	2335	18692
hsa-miR-204-5p	4.69E-06	6.71E-04	98	0	569	2518	37	14352
hsa-miR-425-5p	5.98E-06	8.49E-04	23266	7198	57542	9180	4551	16066
hsa-miR-96-5p	1.34E-05	1.89E-03	4568	898	12421	1545	494	3892
hsa-miR-1301	1.72E-05	2.40E-03	592	0	4777	43	0	188
hsa-miR-335-3p	2.89E-05	4.02E-03	1173	118	4234	5591	440	11145
hsa-miR-183-5p	5.13E-05	7.08E-03	6223	1305	24697	1928	602	5753
hsa-miR-24-2-3p	6.04E-05	8.27E-03	6782	2062	17902	3254	1553	5731
hsa-miR-130b-5p	7.64E-05	1.04E-02	3729	1413	10634	1352	159	2856
hsa-miR-92a-3p	9.93E-05	1.34E-02	182	0	1151	0	0	0
hsa-miR-320a-3p	1.15E-04	1.54E-02	11490	0	29381	2273	0	26275
hsa-miR-130b-3p	1.23E-04	1.64E-02	550	204	1683	246	110	579
hsa-miR-145-5p	1.47E-04	1.94E-02	256	9	603	877	202	2457
hsa-miR-126-3p	1.55E-04	2.03E-02	44031	16691	108893	134103	29907	285350
hsa-miR-139-5p	1.57E-04	2.04E-02	693	181	2537	6623	307	23210
hsa-let-7a-3p	1.59E-04	2.05E-02	175	0	428	41	0	326
hsa-miR-489-5p	1.59E-04	2.05E-02	8	0	63	118	0	483
hsa-miR-362-5p	1.69E-04	2.15E-02	2216	1186	5092	1006	297	2303
hsa-miR-429-5p	1.91E-04	2.40E-02	6416	1486	23455	2082	406	8716
hsa-miR-19b-3p	2.43E-04	3.04E-02	569	0	3214	0	0	0
hsa-miR-362-3p	2.56E-04	3.17E-02	1043	565	2168	448	43	845
hsa-miR-320a-5p	2.72E-04	3.34E-02	1354	0	17859	4369	0	15247
hsa-miR-337-5p	3.44E-04	4.19E-02	386	9	961	1309	334	4086
hsa-miR-143-5p	3.91E-04	4.73E-02	43060	3104	99397	102717	29650	36464

^a^Normalized miRNA reads from each sample were obtained after quantile-quantile scaling. The raw p-value of each miRNA was calculated using the log_10_-scaled miRNA expression data after quantile-quantile scaling and was adjusted using the Holm step down procedure. Relative expression levels are shown.

### Polycistronic miRNA clusters harboring differentially expressed miRNAs in triple-negative breast cancer

The miRNAs residing closely in the same intron, exon, or intergenic region of the human genome were shown to have similar expression patterns in our data. A total of seven miRNA clusters in the human genome were found to be the genomic loci of deregulated miRNAs in triple-negative breast cancer (Table 3). Three miRNA members, including miR-143-5p, miR-145-3p, and miR-145-5p, in the miR-143-145 cluster located in the intergenic region at 5q32 were all significantly down-regulated (*p* <0.05; fold change 2.4-3.4). In addition, miR-497-5p and miR-195-5p, in the miR-497-195 cluster located in the intronic region of *MIR497HG* at 17p13.1, were both significantly down-regulated (*p* <0.05; fold change 3.0-3.3) in triple-negative breast cancer. On the other hand, five other miRNA clusters were found to comprise 24 up-regulated miRNAs in triple-negative breast cancer. These overexpressed miRNAs include four miRNAs (*p* <0.05; fold change 1.9-3.0) in the miR-17-92 cluster, five miRNAs (*p* <0.05; fold change 2.5-3.8) in the miR-183-182 cluster, four miRNAs (*p* <0.05; fold change 1.7-3.1) in the miR-200b-429 cluster, three miRNAs (*p* <0.05; fold change 2.2-3.6) in the miR-301b-130b cluster, and eight miRNAs (*p* <0.05; fold change 2.0-2.6) in the miR-532-502 cluster.

**Table 3:** **Polycistronic miRNA clusters harboring deregulated miRNAs between triple-negative breast cancers and adjacent normal tissues** ^**a**^

**miRNA**	***p*** **value**	**Triple-negative breast cancer**			**Adjacent normal tissue**			**Fold change** ^**b**^ **in expression**
		**Mean**	**Range**		**Mean**	**Range**		
		**Normalized**	**Min**	**Max**	**Normalized**	**Min**	**Max**	
**Down-regulated miRNAs in triple-negative breast cancer**								
**miR-143-145 cluster (Chromosome 5 q32, intergenic)**								
hsa-miR-143-5p	3.91E-04	43060	3104	99397	102717	29650	364644	2.4
hsa-miR-145-3p	7.36E-03	128335	8310	287564	404278	43410	1212945	3.2
hsa-miR-145-5p	1.47E-04	256	9	603	877	202	2457	3.4
**miR-497-195 cluster (Chromosome 17 p13.1, MIR497HG intron 1)**								
hsa-miR-497-5p	2.43E-06	1980	354	6215	6520	2335	18692	3.3
hsa-miR-195-5p	3.18E-03	239	52	504	721	128	2492	3.0
**Up-regulated miRNAs in triple-negative breast cancer**								
**miR-17-92 cluster (Chromosome 13 q31.3,** ***C13orf25*** **intron 3)**								
has-miR-17-3p	2.66E-02	40467	7676	137962	21080	5047	67282	1.9
has-miR-18a-3p	1.43E-02	6362	904	26300	2141	0	11488	3.0
has-miR-19b-3p	9.93E-05	569	0	3214	0	0	0	N/A
has-miR-92a-3p	2.43E-04	182	0	1151	0	0	0	N/A
**miR-183-182 cluster (Chromosome 7 q32.2, intergenic)**								
hsa-miR-183-3p	5.42E-03	160	29	431	63	0	155	2.5
hsa-miR-183-5p	5.13E-05	6223	1305	24697	1928	602	5753	3.2
hsa-miR-96-3p	2.70E-03	65	0	163	26	0	106	2.5
hsa-miR-96-5p	1.34E-05	4568	898	12421	1545	494	3892	3.0
hsa-miR-182-5p	4.76E-03	13189	2441	40871	3501	7	7623	3.8
**miR-200b-429 cluster (Chromosome 1 p36.33, intergenic)**								
hsa-miR-200b-3p	2.65E-02	694	156	2348	398	35	1061	1.7
hsa-miR-200a-3p	2.46E-02	77	0	362	40	0	296	1.9
hsa-miR-200a-5p	1.25E-03	46665	1800	160398	15886	2412	46254	2.9
hsa-miR-429-5p	1.91E-04	6416	1486	23455	2082	406	8716	3.1
**miR-301b-130b cluster (Chromosome 22 q11.21,** ***PPIL2*** **exon 2)**								
hsa-miR-301b-5p	1.41E-03	323	103	1483	89	0	244	3.6
hsa-miR-130b-3p	1.23E-04	550	204	1683	246	110	579	2.2
hsa-miR-130b-5p	7.64E-05	3729	1413	10634	1352	159	2856	2.8
**miR-532-502 cluster (Chromosome X p11.23,** ***CLCN5*** **intron 3)**								
hsa-miR-532-5p	1.58E-02	514	125	2731	253	31	990	2.0
hsa-miR-188-3p	2.72E-02	1159	509	5061	520	0	1258	2.2
hsa-miR-362-3p	2.56E-04	1043	565	2168	448	43	845	2.3
hsa-miR-362-5p	1.69E-04	2216	1186	5092	1006	297	2303	2.2
hsa-miR-501-3p	2.13E-02	335	125	1439	143	0	258	2.3
hsa-miR-660-3p	3.00E-02	11371	5546	23930	4438	0	9196	2.6
hsa-miR-502-3p	1.01E-02	144	60	339	68	0	129	2.1
hsa-miR-502-5p	2.55E-02	354	98	717	174	0	351	2.0

^a^Normalized miRNA reads from each sample were obtained after quantile-quantile scaling. The *p*-value is calculated using the log_10_-scaled miRNA expression data without the Holm adjustment. Relative expression levels are shown.

^b^Fold change was obtained by comparing the mean normalized miRNA expression reads between the triple-negative breast cancers and adjacent normal tissues.

### Quantitative RT-PCR validations of the deregulated miRNAs

**Figure 2:**
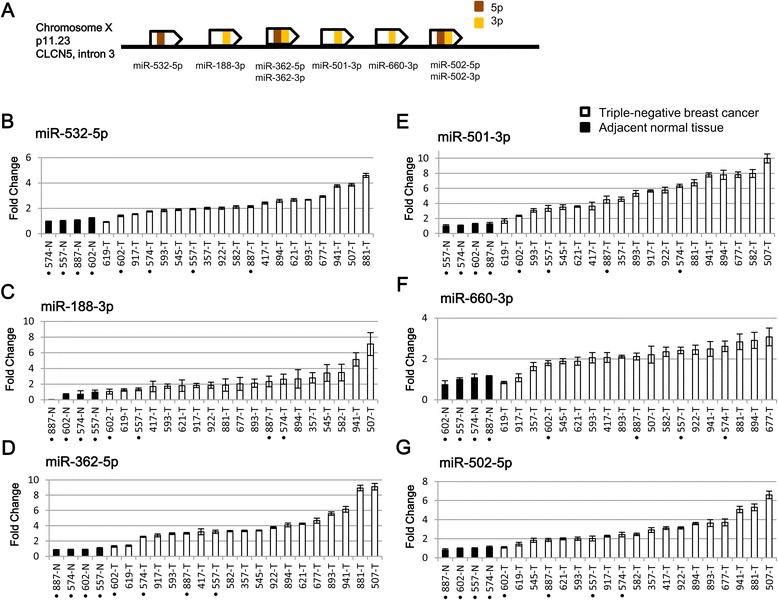
**RT-PCR validations of six differentially expressed miRNAs in the miR-532-502 cluster in triple-negative breast cancer.** **(A)** A conceptual diagram illustrates the genomic loci harboring the 5p miRNA forms (brown) and 3p forms (orange) in the miR-532-502 cluster. The names of specific miRNA forms are provided under each locus. Relative expression levels of **(B)** miR-532-5p, **(C)** miR-188-3p, **(D)** miR-362-5p, **(E)** miR-501-3p, **(F)** miR-660-3p, and **(G)** miR-502-5p in triple-negative breast cancer tissues (n = 19) and adjacent normal tissues (n = 4) are shown. In each panel, paired tissue samples from the same patient are indicated with the same digits in the sample ID. The miRNA expression level in 557-N served as the calibrator control. All six miRNAs examined in the miR-532-502 cluster were markedly up-regulated in triple-negative breast cancer. Each bar represents the mean of triplicate measurements ± the SD.

To confirm the results obtained from the sequencing data, quantitative RT-PCR validations were carried out to examine the expression levels of the miRNAs residing in the miR-532-502 cluster, the miR-301b-130b cluster, and the miR-497-195 cluster in 19 triple-negative breast cancers and 4 adjacent normal tissues using the comparative C_T_ method [13]. A conceptual diagram that depicts the specific 5p/3p miRNA forms located at each locus in the miR-532-502 cluster is shown in Figure 2A. miR-532-5p (Figure 2B; mean fold change 2.4), miR-188-3p (Figure 2C; mean fold change 2.5), miR-362-5p (Figure 2D; mean fold change 4.0), miR-501-3p (Figure 2E; mean fold change 5.3), miR-660-3p (Figure 2F; mean fold change 2.2), and miR-502-5p (Figure 2G; mean fold change 3.0) were all markedly up-regulated in the triple-negative breast cancers compared to the normal breast tissue controls.

**Figure 3:**
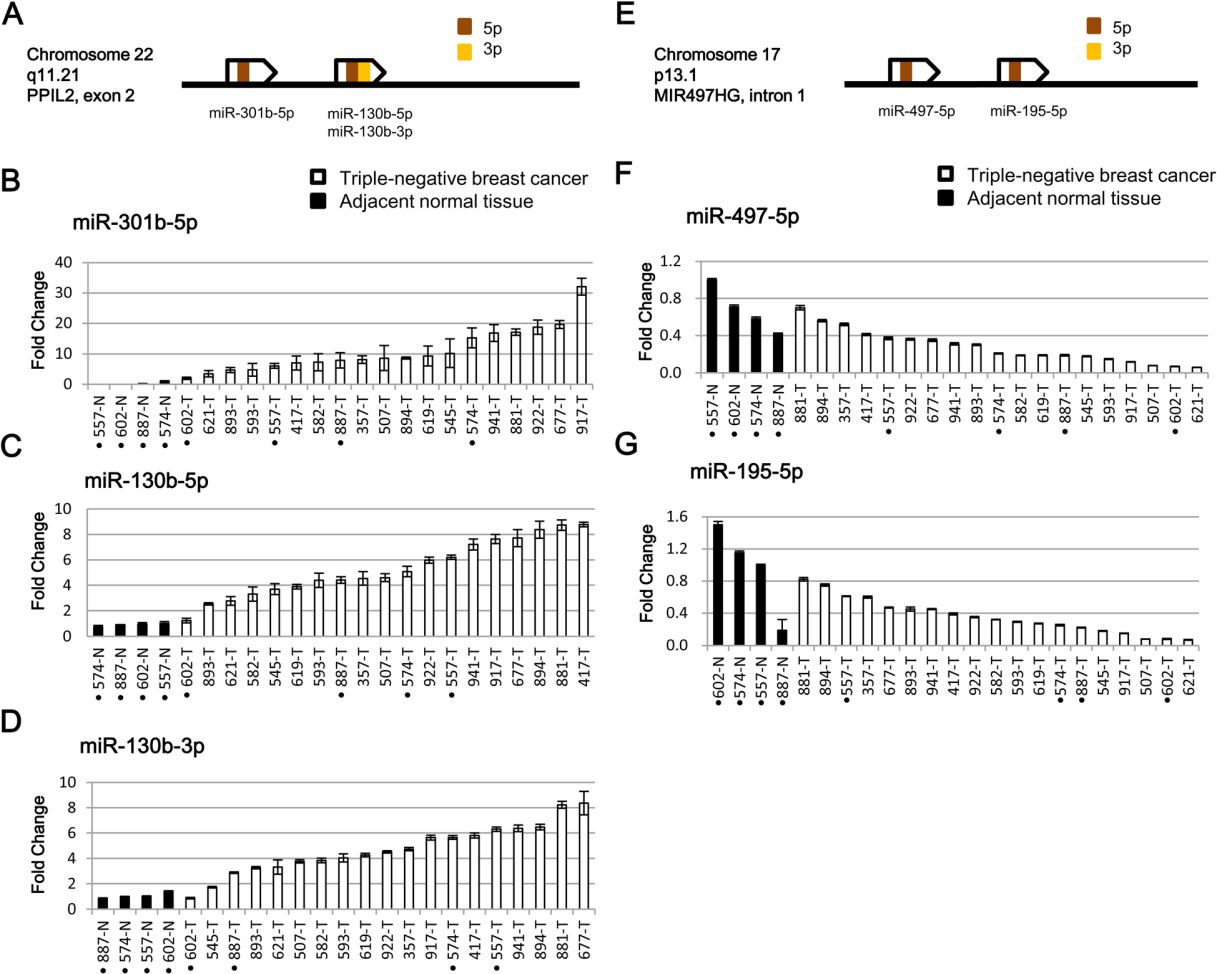
**RT-PCR validations of the differentially expressed miRNAs in the miR-301b-130b cluster and the miR-497-195 cluster. (A)** A conceptual diagram illustrates the genomic loci harboring the 5p miRNA forms (brown) and 3p forms (orange) in the miR-301b-130b cluster. Relative expression levels of **(B)** miR-301b-5p, **(C)** miR-130b-5p, and **(D)** miR-130b-3p in triple-negative breast cancer tissues (n = 19) and adjacent normal tissues (n = 4) are shown. All three miRNAs in the miR-301b-130b cluster were markedly up-regulated in triple-negative breast cancer. **(E)** A conceptual diagram illustrates the genomic loci harboring the 5p miRNA forms (brown) in the miR-497-195 cluster. Relative expression levels of **(F)** miR-497-5p and **(G)** miR-195-5p in 23 samples are shown. Both miRNAs in the miR-497-195 cluster were down-regulated in triple-negative breast cancer. Each bar represents the mean of triplicate measurements ± the SD.

Figure 3A shows the specific miRNA forms at each locus in the miR-301b-130b cluster. Quantitative RT-PCR data confirmed that miR-301b-5p, miR-130b-5p, and miR-130b-3p were all up-regulated in 19 triple-negative breast cancer samples (Figure 3B-D, respectively). Two 5p miRNA forms, miR497-5p and miR-195-5p, located at two different loci in the miR-497-195 cluster are shown in Figure 3E. Compared with the normal breast tissue controls, the expression levels of miR497-5p were markedly down-regulated (Figure 3F; mean fold change 0.3) in triple-negative breast cancers. Furthermore, miR-195-5p in the same cluster was down-regulated (Figure 3G; mean fold change 0.4) in triple-negative breast cancers in the quantitative RT-PCR validations.

### miR-130b-5p directly silences endogenous *CCNG2* expression levels in MDA-MB-231 cells

**Figure 4:**
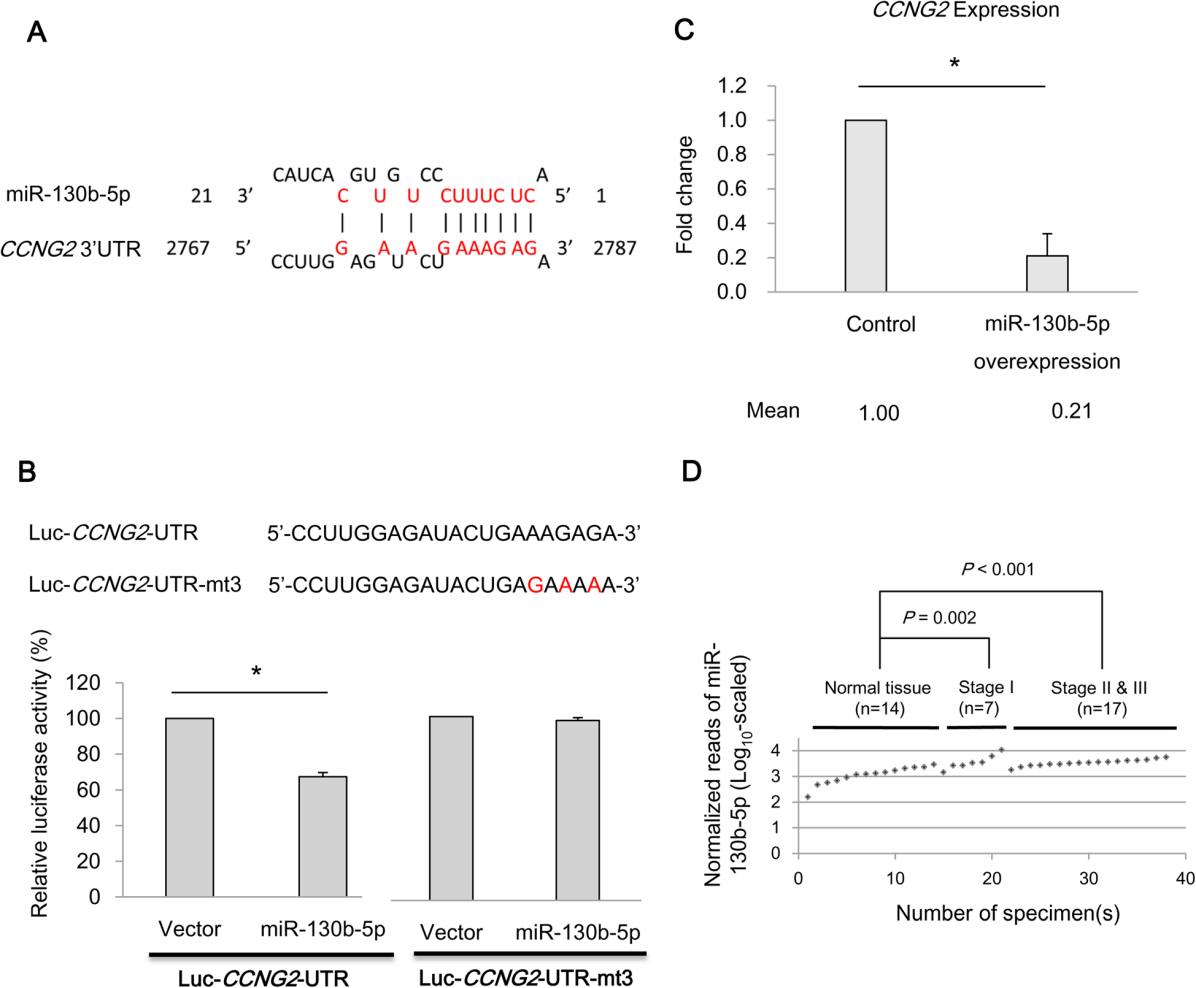
**miR-130b-5p directly silences** ***CCNG2*** **expression in triple-negative breast cancer cells. (A)** Schematic diagram of the putative miR-130b-5p binding site in the 3′-UTR of *CCNG2*. The possible binding sequence of miR-130b-5p with the 3′-UTR of *CCNG2* is shown in red. **(B)** Luciferase assays of *CCNG2* in miR-130b-5p-overexpressed HEK-293 T cells. Two luciferase reporter constructs, Luc-*CCNG2*-UTR and Luc-*CCNG2*-UTR-mt3, were made to test whether miR-130b-5p directly targets the putative binding site in the 3′-UTR of *CCNG2*. The mutated nucleotides are indicated in red. Compared with the negative control, overexpression of miR-130b-5p was shown to significantly inhibit luciferase reporter activity with Luc-*CCNG2*-UTR, but not with Luc-*CCNG2*-UTR-mt3. Each bar represents the mean of triplicate measurements ± the SD; *p <0.05. **(C)** Endogenous expression analysis of *CCNG2* in MDA-MB-231 cells transfected with lentiviral vectors containing miR-130b-5p precursor sequences. The endogenous expression levels of *CCNG2* were significantly repressed after the forced expression of miR-130b-5p in MDA-MB-231 cells. **(D)** Comparisons of miR-130b-5p expression in normal breast tissues (n = 14), early-stage triple-negative breast cancers (n = 7), and advanced-stage triple-negative breast cancers (n = 17). MiRNA expression reads were normalized and analyzed in log_10_ scale. miR-130b-5p expression was significantly associated with both early-stage and advanced-stage triple-negative breast cancers.

To understand how these deregulated miRNAs might function in triple-negative breast cancer, we performed target prediction of the miRNAs as described in the Methods section. Among those putative target candidates, five potential tumor suppressors, including *CCNG2*, *FOXP1*, *NRG1*, *LRIG1*, and *TNFSF10*, were shown to have a direct binding site with miR-130b-5p. We were particularly interested in *CCNG2*, because it was a crucial regulatory gene in cell cycle control [14,15]. Previous studies have functionally linked the decrease or complete loss of *CCNG2* in human cancer to uncontrolled cell proliferation [16,17]. We thus further characterized the regulatory mechanism of miR-130b-5p in relation to *CCNG2* in cells. A schematic diagram of the putative miR-130b-5p binding site in the 3′-UTR of *CCNG2* is shown in Figure 4A. Two luciferase reporter constructs, Luc-*CCNG2*-UTR and Luc-*CCNG2*-UTR-mt3, were made to test whether miR-130b-5p might directly target *CCNG2* in the putative binding site. The possible binding sequences in the 3′-UTR of *CCNG2* were carried by the *CCNG2* gene reporter, Luc-*CCNG2*-UTR. The other luciferase reporter construct, Luc-*CCNG2*-UTR-mt3, was made to carry three mutated nucleotides at the putative binding site to disrupt binding of miR-130b-5p to *CCNG2*. The luciferase reporter assays revealed that miR-130b-5p significantly reduced the luciferase activity of the wild-type *CCNG2* gene reporter, but not that of the site-directed mutant (Figure 4B), suggesting that miR-130b-5p had a suppressive effect at the predicted binding site in the 3′-UTR of *CCNG2*.

Next, we examined whether miR-130b-5p might repress endogenous *CCNG2* expression in triple-negative breast cancer cells. Overexpression of miR-130b-5p was carried out in MDA-MB-231 cells using plasmid DNA transfection of a lentiviral vector containing the precursor sequence of miR-130b-5p. Quantitative RT-PCR data confirmed that the endogenous expression levels of *CCNG2* were significantly repressed in MDA-MB-231 cells after overexpression of miR-130b-5p (Figure 4C). Compared with negative controls, a marked reduction (>50%) in *CCNG2* expression level was observed in the miR-130b-5p-overexpressed MDA-MB-231cells.

The functional relevance of miR-130b-5p in cell cycle regulation with triple-negative breast cancer cells was analyzed in this study. After overexpression of miR-130b-5p, MDA-MB-231 cells showed significant alterations (*p* <0.05) in cell cycle profile (Additional file 2). A significant decrease in G1 phase and increases in S and G2/M phases were observed with the miR-130b-5p-overexpressed MDA-MB-231cells in the cell cycle analysis. These results strongly support that miR-130b-5p plays a role in cell cycle progression through suppression of *CCNG2* expression in triple-negative breast cancer.

We then investigated an association between miR-130b-5p expression and malignancy of triple-negative breast cancer from the sequencing data in our cohort. Sequencing data of miR-130b-5p expression were shown to be significantly associated with tumor progression in both early-stage triple-negative breast cancers (*p* = 0.002; n = 7) and advanced stage triple-negative breast cancers (*p* <0.001; n = 17) in Figure 4D. The expression levels of *CCNG2* in our cohort of 38 samples were also measured using Agilent microarray (Human 1A v2). A direct correlation between high miR-130b-5p expression (*p* <0.001; fold change 2.8) and low *CCNG2* expression (*p* <0.001; fold change 0.5) was observed in our triple-negative breast cancer samples as compared with normal breast tissue controls (Additional file 3A and B).

## Discussion

Sequencing data of miRNA expression reads from 24 triple-negative breast cancers and 14 adjacent normal tissues were analyzed in this study. Deregulated miRNAs were identified from the statistical analyses and a panel of the top 25 deregulated miRNAs was found to be an effective discriminator between triple-negative breast cancers and adjacent normal tissues. Deep sequencing technology allowed us to generate a comprehensive insight into the cellular transcriptome in triple-negative breast cancer that led to the identification of many more deregulated miRNAs not described in previous studies [18,19]. For example, aberrant expression from the miR-532-502 cluster in triple-negative breast cancer was first documented in this study. Interestingly, the expression level of the tumor suppressor gene *RUNX3* was found to be inversely correlated with that of miR-532-5p from the miR-532-502 cluster in primary melanomas [20].

A number of the validated target genes of the deregulated miRNAs in our findings were shown to be involved in human cancer signaling cascades (Additional file 4). For example, we observed that both miR-143-5p and miR-145-5p in the miR-143-145 cluster were down-regulated in triple-negative breast cancer. Down-regulation of these two miRNAs was previously described in lung cancer, colon cancer, and bladder cancer [21-23]. The proto-oncogene c-Myc was a direct target for miR-145-5p, and introduction of miR-145-5p repressed c-Myc expression and tumor growth both *in vitro* and *in vivo* [11]. The insulin receptor substrate-1 (*IRS-1*), previously known as a major docking protein for both the type 1 insulin-like growth factor receptor and the insulin receptor in cancer cell growth and proliferation signaling, was also a validated target of miR-145-5p [24]. In addition, Sachdeva *et al.* reported that cell invasion ability was significantly inhibited by miR-145-5p, in part due to the silencing of the metastasis gene *MUC1* [25].

Deregulation of the miR-497-195 cluster has been previously addressed in breast cancer. Li *et al.* described that miR-497-5p and miR-195-5p in this cluster were both down-regulated in human breast cancer tissues and cell lines [26]. However, the study did not specifically determine whether the deregulation of these two miRNAs observed in breast cancer cell lines was also observed in triple-negative breast cancer tissues. Our results provide evidence showing *in vivo* that both miR-497-5p and miR-195-5p are down-regulated in triple-negative breast cancer tissues. Furthermore, the methylation state of CpG islands in the promoter region upstream of the miR497-195 cluster was responsible for the down-regulation of those two miRNAs in breast cancer, and direct targets of miR-195-5p included *CCND1* and *RAF1* [26]. Moreover, introduction of miR-195-5p was shown to inhibit cancer cell colony formation *in vitro* [26] and tumor development in nude mice [27], suggesting that ectopic expression of miR-195-5p may be involved in the tumorigenesis of breast cancer.

We were able to identify *CCNG2* as a direct target of miR-130b-5p in triple-negative breast cancer. Luciferase reporter assays revealed that miR-130b-5p-mediated repression of *CCNG2* is dependent on the sequence of the 3′-UTR. *CCNG2* is known as a negative regulator of cell cycle progression. Previous microarray analyses have shown that elevated CCNG2 expression induces cell cycle arrest during responses to various types of growth inhibitory effects, such as hypoxia, oxidative stress, and heat shock [17,28,29]. The CCNG2 protein directly interacts with the catalytic subunit of protein phosphatase 2A to form active complexes that inhibit cell cycle progression [15]. Increasingly, the evidence suggests that CCNG2 is crucially involved in human cancer signaling pathways [30-32]. For example, *CCNG2* was a primary target gene of estrogen-occupied estrogen receptor and that its expression was rapidly down-regulated by estrogens in MCF-7 breast cancer cells [31]. Moreover, *CCNG2* promoter activity was found to be regulated by Nodal signaling in ovarian cancer cells and silencing of CCNG2 expression significantly increased cell proliferation [32].

Gene expression levels of *CCNG2* between triple-negative breast cancers and normal breast tissues were further investigated using a published microarray dataset [GEO:GSE53752]. The gene expression levels of *CCNG2* in triple-negative breast cancers (n = 51) were significantly lower (*p* <0.001; fold change 1.9) than those in normal breast tissues (n = 25) in the microarray data (Additional file 5). Of added interest, recently *CCNG2* was found to be an important prognostic factor for triple-negative breast cancer patients. In an analysis of a cohort of 250 primary triple-negative breast cancer samples from eight clinically annotated gene expression datasets, triple-negative breast cancer patients with low expression levels of *SHARP1* and *CCNG2* had a significantly higher probability of developing metastases and of reduced survival [33]. It is thus important that we identified *CCNG2* as a direct target of miR-130b-5p. The ability of miR-130b-5p to repress CCNG2 expression may enhance malignancy by accelerating cell cycle transition in triple-negative tumor cells.

## Conclusions

Our work clearly depicts the global miRNA regulatory characteristics in triple-negative breast cancer. The 25-miRNA signature determined in this study may be used as a functional tool to distinguish triple-negative breast cancer tissues from normal breast tissues. Seven polycistronic miRNA clusters preferentially harboring deregulated miRNAs were identified in triple-negative breast cancer. Moreover, we extended the current knowledge of microRNA regulatory network by showing that miR-130b-5p in the miR-301b-130b cluster directly silences *CCNG2* in triple-negative breast cancer. It would be interesting to test whether this novel regulatory mechanism of miR-130b-5p and its *CCNG2* target in triple-negative breast cancer may be involved in other malignancies as well. Our findings not only provide insight into the miRNA regulatory mechanisms in triple negative breast cancer, but also shed light on the identification of potential therapeutic targets for this disease.

## Methods

### Breast cancer and normal tissue samples

Twenty-four triple-negative breast cancer samples and 14 adjacent normal tissue samples were collected from breast cancer patients during surgeries at National Taiwan University Hospital (NTUH, Taipei, Taiwan). All triple-negative breast cancer samples were invasive ductal carcinomas and were negative in immunohistochemical analyses of ER, PR, and HER2. AJCC/UICC TNM staging system was used for tumor classification. Treatment of each patient followed the National Comprehensive Cancer Network (http://www.nccn.org/) guidelines. All samples were neoadjuvant-free and were collected before systemic chemotherapy treatments. Written informed consent was obtained from each patient who participated in this study. All human tissues used in this study were approved by the institutional review board at NTUH.

### Small RNA library preparation

Total RNA was extracted from each sample for the preparation of a small RNA library. The small RNA library was constructed from total RNA using the SOLiD Total RNA-Seq Kit (Applied Biosystems, Foster City, CA, USA). Integrity of each small RNA library was examined using an RNA 6000 Nano Chip (Agilent, Santa Clara, CA, USA), a Small RNA Chip (Agilent), and the Bioanalyzer (Agilent) according to the manufacturer’s instructions.

### Deep sequencing experiments

Upon completion of PCR amplification, the small RNA libraries were purified using the SOLiD Library Micro Column Purification Kit (Applied Biosystems) and hybridized to the template beads using the SOLiD EZ bead system (Applied Biosystems). The template beads were amplified and deposited onto a tray for small RNA ligation sequencing by the SOLiD 4 System (Applied Biosystems). The sequencing data were uploaded to the Gene Expression Omnibus (GEO) with an accession number of GSE40049.

### Sequence alignment of miRNA reads

All reads obtained from ligation sequencing were first screened to filter out the reads containing ribosomal RNA, transfer RNA, and adaptor sequences. The remaining reads were then aligned to the human miRNA reference (miRBase v17.0) and the human genome reference (RefSeq Hg19) using the Small RNA Analysis Tool (Applied Biosystems). In the sequence alignment, only one mismatch was allowed for the first 16 bases of a miRNA read. The maximum number of permitted mismatches of a miRNA read was set at 4. Those reads that were not uniquely mapped to the miRBase reference were disregarded to eliminate ambiguous alignments.

### Statistical analyses

The quantile-quantile scaling method [34] was performed for the normalization of miRNA expression reads in log_10_-scale. All miRNA expression reads in each dataset were linearly scaled to fit into a miRNA expression reference made of the mean expression value of each miRNA from 38 samples. Principal component analysis (PCA) was performed to analyze the miRNA expression profiles between triple-negative breast cancers and adjacent normal tissues with the Partek Genomics Suite (Partek Incorporated, St. Louis, MO, USA). Significant differences in expression of miRNAs from the triple-negative breast cancers and adjacent normal tissues were identified using two-tailed Student’s t-tests. The Holm step down procedure was used to counteract multiple comparisons. A *p*-value of <0.05 was considered significant. The miRNAs with a fold change >2 and mean expression difference >100 reads between the two groups were investigated in the hierarchical clustering analysis using Genesis software (version 1.7.5).

### Quantitative RT-PCR validation of miRNA expression

Differentially expressed miRNAs identified from our sequencing data were validated using quantitative RT-PCR in 19 triple-negative breast cancer samples and 4 adjacent normal tissue samples. Total RNA was extracted from each sample and then reverse transcribed into miRNA-specific cDNA following the standard protocol of the TaqMan MicroRNA Reverse Transcription Kit (Applied Biosystems). Relative quantification of miRNA expression in each sample was obtained using the comparative threshold (C_T_) method [13]. Expression of U6 small nuclear RNA was used as the endogenous control.

### Putative target prediction

Potential mRNA target genes of a miRNA were searched using the miRanda [35] and Diana [36] target prediction algorithms. Putative target candidates having complementary base-pairing matches in the 3′-UTR for the indicated miRNA seed region were obtained. Biological functions associated with the target genes were investigated using Ingenuity Pathway Analysis software (Ingenuity Systems, Redwood City, CA, USA).

### Experimentally validated miRNA target genes

Experimentally validated miRNA target genes were retrieved from TarBase [37] and miRecords [38] using the miRSystem search engine [39]. The molecular pathways encompassing the validated target genes were investigated using Ingenuity Pathway Analysis software.

### Cell lines

MDA-MB-231 and HEK-293 T were obtained from Bioresource Collection and Research Center (Taiwan). The cell lines were tested and authenticated by Genelabs Life Science (Taiwan) using STR-PCR profiling.

### Vectors

miR-130b-5p was cloned into a lentiviral vector PreMiR-130b (System Biosciences, Mountain View, CA, USA) that was used to overexpress the miRNA in MDA-MB-231 cells. Expression of miR-130b-5p was verified and quantified using KAPA PROBE Fast qPCR Master Mix (Kapa Biosystems, Boston, MA, USA), and the LightCycler 480 System (Roche, Basel, Switzerland).

A *CCNG2* luciferase reporter construct was made by introducing the *CCNG2* 3′-UTR carrying a predicted miR-130b-5p binding site (5′-CCTTGGAGATACTGAAAGAGA-3′) into the pmirGLO control vector (Promega, Madison, WI, USA). Site-directed mutagenesis of the putative miR-130b-5p binding site was made using a facile PCR procedure [40]. All PCR products were verified by DNA sequencing before use.

### Luciferase assay

Luciferase assays were performed with HEK-293 T cells using the Dual-Glo® Luciferase Assay System (Promega). Cells were transfected with PreMiR-130b lentiviral vectors using TransIT®-2020 transfection reagent (Mirus Bio, Madison, WI, USA). Forty-eight hours after transfection, the cells were then harvested and lysed for the luciferase assay. Renilla luciferase signals were used for normalization according to the manufacturer’s protocol.

### Cell cycle analysis

3*×*10^4^ cells of MDA-MB-231 cells were seeded in a 24-well plate. miR-130b-5p or empty vector (control) were overexpressed in the MDA-MB-231 cells. Cell synchronization was performed using double thymidine block. 2 mM of thymidine was added into cells and cells were incubated at 37°C for 16 hours. To remove thymidine, cells were washed with PBS and incubated with fresh media at 37°C for 8 hours. 2 mM of thymidine was added into cells and cells were incubated at 37°C for 16 hours. To release the cells from thymidine block, cells were washed with PBS and incubated with fresh media and collected after 14 hours. The cells were harvested by trypsinization and washed twice with cold PBS. The cells were fixed by 0.5 mL of cold 95% ethanol and were kept at -20°C overnight. The ethanol was removed and cells were washed twice with PBS. The cells were resuspended in 500 μL of PI solution (10 μg/mL of propidium iodide, 0.2 mg/mL of RNaseA and 0.1% Triton X-100). Cell cycle profile was analyzed by fluorescence-activated cell sorter (FACS) analysis.

### Gene expression microarray experiments

Gene expression data of *CCNG2* in our cohort of 38 samples (14 normal breast tissues and 24 triple-negative breast cancer samples) were analyzed using Agilent Human 1A (version 2) microarray platform. Microarray experiments were performed following the manufacturer’s instructions. Microarray data were normalized using Quantile normalization before statistical analyses.

### Online supporting information

The sequencing data were uploaded to the Gene Expression Omnibus (GEO) with an accession number of GSE40049.

## Additional files

Additional file 1:
**Raw miRNA reads from triple-negative breast cancers (N = 24) and adjacent normal tissues (N = 14) obtained using the high-throughput sequencer.**
Additional file 2:
**Cell cycle analysis showed significant alterations in cell cycle profile in MDA_MB-231 cells after miR-130b-5p overexpression.** MDA-MB-231 cells were transfected with empty vectors (control) or vectors containing miR-130b-5p precursor sequence. Compared with vector controls, there was a significant decrease with the cell population in G1 phase along with increases in S and G2/M phases in the miR-130b-5p-overexpressed MDA_MB-231 cells. The data were analyzed using paired t-test. **p* <0.05. Bars, ±SD. Additional file 3:
**Expression levels of miR-130b-5p and** ***CCNG2*** **were inversely regulated between normal breast tissues (n = 14) and triple-negative breast cancers (n = 24) in our cohort of 38 samples.** (A) Normalized deep-sequencing reads of miR-130b-5p in triple-negative breast cancers and normal breast tissue controls are shown in the box plots. miR-130b-5p expression was significantly (*p* <0.001) up-regulated in triple-negative breast cancers. The *p* value was calculated with the normalized log_10_-scaled miRNA reads using the parametric t-test. (B) Agilent microarray data of *CCNG2* expression in triple-negative breast cancers and normal breast tissue controls in our cohort are shown in the box plots. Microarray data were normalized using quantile normalization before the statistical analyses. *CCNG2* was significantly (*p* <0.001) down-regulated in triple-negative breast cancers. Fold change was calculated using the mean expression data from the two groups. The *p* value was obtained using the parametric t-test. Additional file 4:
**Experimentally validated miRNA-target gene relationships of the deregulated miRNAs in triple-negative breast cancer.** The associations between miRNAs and human cancer were retrieved from curated databases. Additional file 5:
**Comparison of** ***CCNG2*** **expression levels between normal breast tissues (n = 25) and triple-negative breast cancers (n = 51) from a published microarray dataset [GEO:GSE33926].** The microarray data were obtained using the Agilent Human 1A (version 2) platform. Quantile normalization was performed on the microarray data before the parametric t-test analysis. The *p-*value was adjusted using Bonferroni correction for multiple comparisons. The microarray data revealed that, compared with normal breast tissues, *CCNG2* was significantly down-regulated in triple-negative breast cancers.

## Abbreviations

miRNA: MicroRNA
CCNG2: Cyclin G2
ER: Estrogen receptor
PR: Progesterone receptor
HER2: Human epidermal growth factor receptor 2
mRNA: messenger RNA
UTR: Untranslated region
RT-PCR: Reverse transcription PCR
NTUH: National Taiwan University Hospital
IHC: Immunohistochemical
GEO: Gene Expression Omnibus
PCA: Principal component analysis
SD: Standard deviation
