# Supplementary material for: Deregulated microRNAs in triple-negative breast cancer revealed by deep sequencing
Source: Mol Cancer. 2015 Feb 10;14:36. doi: 10.1186/s12943-015-0301-9 (PMC4351690; doi:10.1186/s12943-015-0301-9)
Supplement: Additional file 1: — Raw miRNA reads from triple-negative breast cancers (N = 24) and adjacent normal tissues (N = 14) obtained using the high-throughput sequencer. [file 12943_2015_301_MOESM1_ESM.doc]

Additional file 1. Raw miRNA reads from triple-negative breast cancers (N = 24) and adjacent normal tissues (N = 14) obtained using the high-throughput sequencer.

| Sample ID | Sample type | Reads (from 5’ arms) | (%) | Reads (from 3’ arms) | (%) | Total reads | (%) |
| --- | --- | --- | --- | --- | --- | --- | --- |
| 291_T | TNBC | 1161255 | (26.8) | 3171863 | (73.2) | 4333118 | (100.0) |
| 357_T | TNBC | 1139981 | (35.2) | 2095672 | (64.8) | 3235653 | (100.0) |
| 417_T | TNBC | 1600779 | (29.5) | 3829783 | (70.5) | 5430562 | (100.0) |
| 452_T | TNBC | 1366063 | (40.9) | 1972123 | (59.1) | 3338186 | (100.0) |
| 477_T | TNBC | 792973 | (34.3) | 1521893 | (65.7) | 2314866 | (100.0) |
| 507_T | TNBC | 1144709 | (35.7) | 2062240 | (64.3) | 3206949 | (100.0) |
| 545_T | TNBC | 513406 | (40.9) | 742296 | (59.1) | 1255702 | (100.0) |
| 557_T | TNBC | 823093 | (20.5) | 3197974 | (79.5) | 4021067 | (100.0) |
| 574_T | TNBC | 695530 | (32.3) | 1457975 | (67.7) | 2153505 | (100.0) |
| 582_T | TNBC | 522500 | (24.9) | 1577594 | (75.1) | 2100094 | (100.0) |
| 593_T | TNBC | 566777 | (22.5) | 1955789 | (77.5) | 2522566 | (100.0) |
| 602_T | TNBC | 215879 | (24.7) | 659142 | (75.3) | 875021 | (100.0) |
| 619_T | TNBC | 1018398 | (26.6) | 2816066 | (73.4) | 3834464 | (100.0) |
| 621_T | TNBC | 1658711 | (42.8) | 2221043 | (57.2) | 3879754 | (100.0) |
| 673_T | TNBC | 159869 | (40.6) | 233436 | (59.4) | 393305 | (100.0) |
| 677_T | TNBC | 409893 | (31.3) | 900210 | (68.7) | 1310103 | (100.0) |
| 881_T | TNBC | 1257332 | (34.7) | 2363906 | (65.3) | 3621238 | (100.0) |
| 887_T | TNBC | 523782 | (45.3) | 632916 | (54.7) | 1156698 | (100.0) |
| 893_T | TNBC | 878872 | (32.6) | 1814636 | (67.4) | 2693508 | (100.0) |
| 894_T | TNBC | 1022675 | (44.7) | 1266586 | (55.3) | 2289261 | (100.0) |
| 917_T | TNBC | 1002537 | (34.7) | 1890117 | (65.3) | 2892654 | (100.0) |
| 918_T | TNBC | 2160099 | (49.5) | 2203709 | (50.5) | 4363808 | (100.0) |
| 922_T | TNBC | 847340 | (32.0) | 1799636 | (68.0) | 2646976 | (100.0) |
| 941_T | TNBC | 1144062 | (33.5) | 2273784 | (66.5) | 3417846 | (100.0) |
| 291_N | Normal | 1710897 | (35.0) | 3182236 | (65.0) | 4893133 | (100.0) |
| 357_N | Normal | 2559351 | (50.2) | 2543617 | (49.8) | 5102968 | (100.0) |
| 477_N | Normal | 1509385 | (59.7) | 1020898 | (40.3) | 2530283 | (100.0) |
| 507_N | Normal | 2210175 | (42.7) | 2969580 | (57.3) | 5179755 | (100.0) |
| 557_N | Normal | 292343 | (35.7) | 525934 | (64.3) | 818277 | (100.0) |
| 574_N | Normal | 358120 | (34.5) | 678690 | (65.5) | 1036810 | (100.0) |
| 582_N | Normal | 669733 | (38.9) | 1052696 | (61.1) | 1722429 | (100.0) |
| 602_N | Normal | 379389 | (28.6) | 947667 | (71.4) | 1327056 | (100.0) |
| 673_N | Normal | 373659 | (39.2) | 578487 | (60.8) | 952146 | (100.0) |
| Continued |  |  |  |  |  |  |  |
| 677_N | Normal | 1863106 | (30.7) | 4210923 | (69.3) | 6074029 | (100.0) |
| 881_N | Normal | 2494338 | (51.2) | 2374136 | (48.8) | 4868474 | (100.0) |
| 887_N | Normal | 555967 | (43.5) | 720850 | (56.5) | 1276817 | (100.0) |
| 918_N | Normal | 3443587 | (43.6) | 4463047 | (56.4) | 7906634 | (100.0) |
| 922_N | Normal | 738581 | (30.3) | 1698272 | (69.7) | 2436853 | (100.0) |

TNBC: triple-negative breast cancer
