# Supplementary material for: Deregulated microRNAs in triple-negative breast cancer revealed by deep sequencing
Source: Mol Cancer. 2015 Feb 10;14:36. doi: 10.1186/s12943-015-0301-9 (PMC4351690; doi:10.1186/s12943-015-0301-9)
Supplement: Additional file 2: — Cell cycle analysis showed significant alterations in cell cycle profile in MDA_MB-231 cells after miR-130b-5p overexpression. MDA-MB-231 cells were transfected with empty vectors (control) or vectors containing miR-130b-5p precursor sequence. Compared with vector controls, there was a significant decrease with the cell population in G1 phase along with increases in S and G2/M phases in the miR-130b-5p-overexpressed MDA_MB-231 cells. The data were analyzed using paired t-test. *p <0.05. Bars, ±SD. [file 12943_2015_301_MOESM2_ESM.doc]

Additional file 2. Cell cycle analysis showed significant alterations in cell cycle profile in MDA_MB-231 cells after miR-130b-5p overexpression. MDA-MB-231 cells were transfected with empty vectors (control) or vectors containing miR-130b-5p precursor sequence. Compared with vector controls, there was a significant decrease with the cell population in G1 phase along with increases in S and G2/M phases in the miR-130b-5p-overexpressed MDA_MB-231 cells. The data were analyzed using paired t-test. *, *p* <0.05. Bars, ±SD.

**
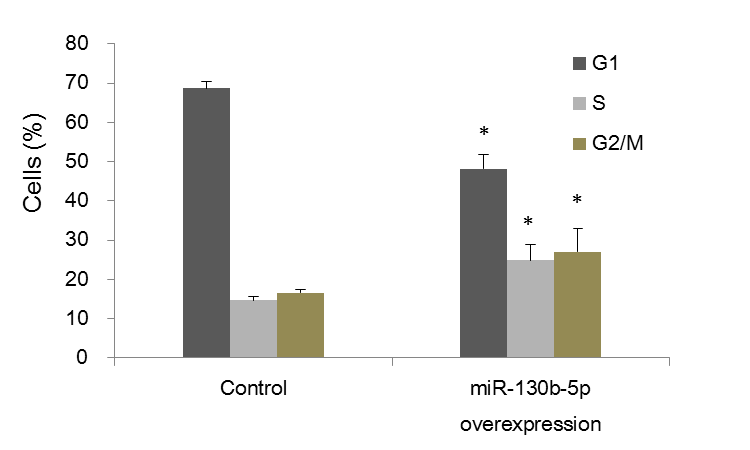
**
