# Supplementary material for: Deregulated microRNAs in triple-negative breast cancer revealed by deep sequencing
Source: Mol Cancer. 2015 Feb 10;14:36. doi: 10.1186/s12943-015-0301-9 (PMC4351690; doi:10.1186/s12943-015-0301-9)

Additional file 3. Expression levels of miR-130b-5p and *CCNG2* were inversely regulated between normal breast tissues (n = 14) and triple-negative breast cancers (n = 24) in our cohort of 38 samples. (A) Normalized deep-sequencing reads of miR-130b-5p in triple-negative breast cancers and normal breast tissue controls are shown in the box plots. miR-130b-5p expression was significantly (*p* <0.001) up-regulated in triple-negative breast cancers. The *p* value was calculated with the normalized log_10_-scaled miRNA reads using the parametric t-test. (B) Agilent microarray data of *CCNG2* expression in triple-negative breast cancers and normal breast tissue controls in our cohort are shown in the box plots. Microarray data were normalized using quantile normalization before the statistical analyses. *CCNG2* was significantly (*p* <0.001) down-regulated in triple-negative breast cancers. Fold change was calculated using the mean expression data from the two groups. The *p* value was obtained using the parametric t-test.


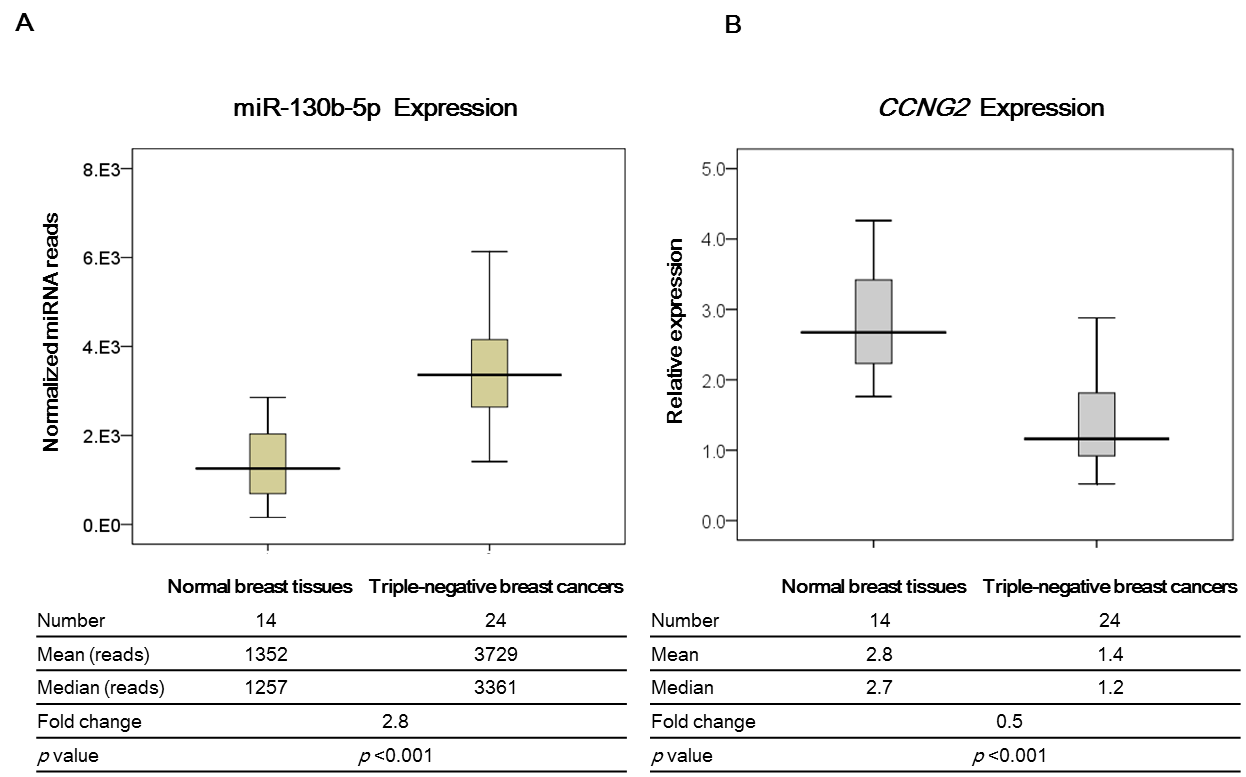

Supplement: Additional file 3: — Expression levels of miR-130b-5p and CCNG2 were inversely regulated between normal breast tissues (n = 14) and triple-negative breast cancers (n = 24) in our cohort of 38 samples. (A) Normalized deep-sequencing reads of miR-130b-5p in triple-negative breast cancers and normal breast tissue controls are shown in the box plots. miR-130b-5p expression was significantly (p <0.001) up-regulated in triple-negative breast cancers. The p value was calculated with the normalized log10-scaled miRNA reads using the parametric t-test. (B) Agilent microarray data of CCNG2 expression in triple-negative breast cancers and normal breast tissue controls in our cohort are shown in the box plots. Microarray data were normalized using quantile normalization before the statistical analyses. CCNG2 was significantly (p <0.001) down-regulated in triple-negative breast cancers. Fold change was calculated using the mean expression data from the two groups. The p value was obtained using the parametric t-test. [file 12943_2015_301_MOESM3_ESM.doc]
