# Supplementary material for: Deregulated microRNAs in triple-negative breast cancer revealed by deep sequencing
Source: Mol Cancer. 2015 Feb 10;14:36. doi: 10.1186/s12943-015-0301-9 (PMC4351690; doi:10.1186/s12943-015-0301-9)
Supplement: Additional file 4: — Experimentally validated miRNA-target gene relationships of the deregulated miRNAs in triple-negative breast cancer. The associations between miRNAs and human cancer were retrieved from curated databases. [file 12943_2015_301_MOESM4_ESM.doc]

Additional file 4. Experimentally validated miRNA-target gene relationships of the deregulated miRNAs in triple-negative breast cancer. The associations between miRNAs and human cancer were retrieved from curated databases.

| miRNA | Experimentally validated target gene | Associated human cancer | Reference |  | | | | |
| --- | --- | --- | --- | --- | --- | --- | --- | --- |
| **Tumor-suppressing miRNAs** | | | |  | | | | |
| hsa-miR-126-3p | *CRKL, IRS1, PIK3R2, VEGFA* | Lung cancer, colon cancer, breast cancer |  |  | | | | |
| hsa-miR-143-5p | *MAPK7* | Colon cancer |  |  | | | | |
| hsa-miR-145-5p | *MYC, FSCN1, CCNA2, MUC1, CLINT1, PPP3CA, FBXO28, ACBD3, USP46, RASA1, CCDC25, IGF-IR, KRT7, RTKN, IRS1* | Bladder cancer, prostate cancer, colon cancer, breast cancer |  |  | | | | |
| hsa-miR-195-5p | *CCND1, RAF1, E2F3, CDK6* | Breast cancer, liver cancer |  |  | | | | |
| hsa-miR-204-5p | *SPDEF, CTSC, ATP2B1, SOX4, FBN2, SHC1, CDC25B, HMGA2, CDH11, SPARC, MMP9, ARPC1B, HOXB7, EFNB1* | Breast cancer, squamous cell carcinoma of the head and neck |  |  | | | | |
| **Oncogenic miRNAs** | | | | |  |  |  |  |
| has-miR-19b-3p | *CTGF, ESR1, PTEN* | Glioblastoma, neuroblastoma, breast cancer |  |  | | | | |
| hsa-miR-183 | *FOXO1* | Endometrial cancer |  |  | | | | |
| hsa-miR-182 | *FOXO1* | Endometrial cancer |  |  | | | | |
| hsa-miR-200 | *FOG2, ZEB1, ZEB2* | Ovarian cancer |  |  | | | | |
| hsa-miR-532-5p | *RUNX3* | Skin cancer |  |  | | | | |
