# Supplementary material for: Deregulated microRNAs in triple-negative breast cancer revealed by deep sequencing
Source: Mol Cancer. 2015 Feb 10;14:36. doi: 10.1186/s12943-015-0301-9 (PMC4351690; doi:10.1186/s12943-015-0301-9)
Supplement: Additional file 5: — Comparison of CCNG2 expression levels between normal breast tissues (n = 25) and triple-negative breast cancers (n = 51) from a published microarray dataset [GEO:GSE33926]. The microarray data were obtained using the Agilent Human 1A (version 2) platform. Quantile normalization was performed on the microarray data before the parametric t-test analysis. The p-value was adjusted using Bonferroni correction for multiple comparisons. The microarray data revealed that, compared with normal breast tissues, CCNG2 was significantly down-regulated in triple-negative breast cancers. [file 12943_2015_301_MOESM5_ESM.doc]

Additional file 5. Comparison of *CCNG2* expression levels between normal breast tissues (n=25) and triple-negative breast cancers (n=51) from a published microarray dataset [GEO:GSE33926]. The microarray data were obtained using the Agilent Human 1A (version 2) platform. Quantile normalization was performed on the microarray data before the parametric t-test analysis. The *p-*value was adjusted using Bonferroni correction for multiple comparisons. The microarray data revealed that, compared with normal breast tissues, *CCNG2* was significantly down-regulated in triple-negative breast cancers.


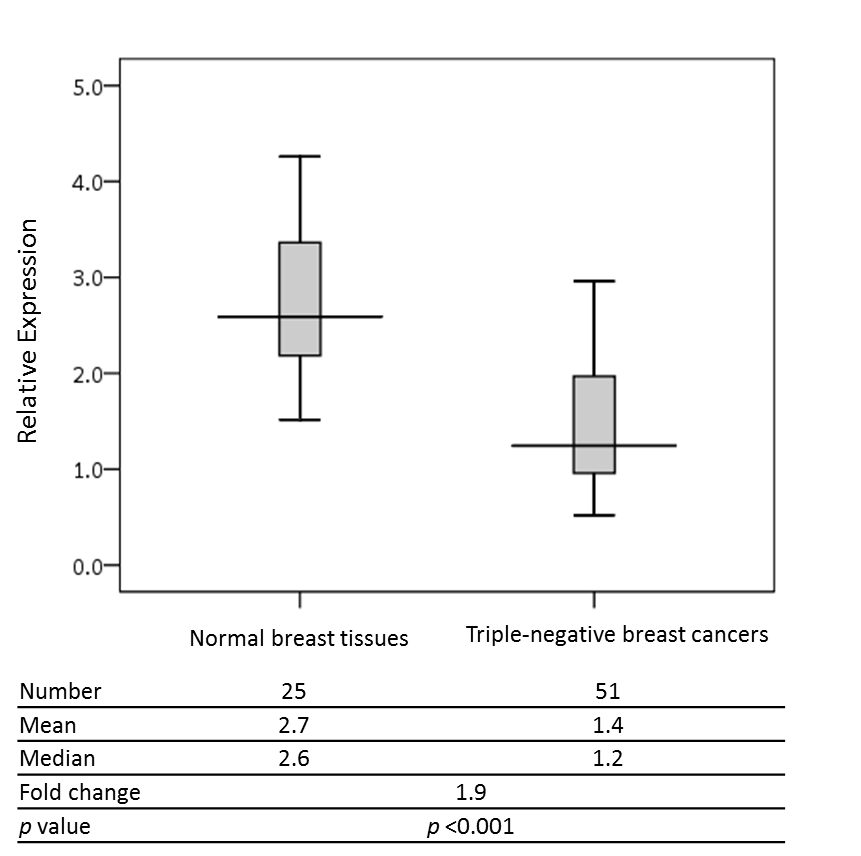


**References**

1. Crawford M, Brawner E, Batte K, Yu L, Hunter MG, Otterson GA, Nuovo G, Marsh CB, Nana-Sinkam SP: **MicroRNA-126 inhibits invasion in non-small cell lung carcinoma cell lines.** *Biochem Biophys Res Commun* 2008, **373:**607-612.

2. Zhang J, Du YY, Lin YF, Chen YT, Yang L, Wang HJ, Ma D: **The cell growth suppressor, mir-126, targets IRS-1.** *Biochem Biophys Res Commun* 2008, **377:**136-140.

3. Guo C, Sah JF, Beard L, Willson JK, Markowitz SD, Guda K: **The noncoding RNA, miR-126, suppresses the growth of neoplastic cells by targeting phosphatidylinositol 3-kinase signaling and is frequently lost in colon cancers.** *Genes Chromosomes Cancer* 2008, **47:**939-946.

4. Zhu N, Zhang D, Xie H, Zhou Z, Chen H, Hu T, Bai Y, Shen Y, Yuan W, Jing Q, Qin Y: **Endothelial-specific intron-derived miR-126 is down-regulated in human breast cancer and targets both VEGFA and PIK3R2.** *Mol Cell Biochem* 2011, **351:**157-164.

5. Akao Y, Nakagawa Y, Naoe T: **MicroRNA-143 and -145 in colon cancer.** *DNA Cell Biol* 2007, **26:**311-320.

6. Sachdeva M, Zhu S, Wu F, Wu H, Walia V, Kumar S, Elble R, Watabe K, Mo YY: **p53 represses c-Myc through induction of the tumor suppressor miR-145.** *Proc Natl Acad Sci U S A* 2009, **106:**3207-3212.

7. Chiyomaru T, Enokida H, Tatarano S, Kawahara K, Uchida Y, Nishiyama K, Fujimura L, Kikkawa N, Seki N, Nakagawa M: **miR-145 and miR-133a function as tumour suppressors and directly regulate FSCN1 expression in bladder cancer.** *Br J Cancer* 2010, **102:**883-891.

8. Wang L, Tang H, Thayanithy V, Subramanian S, Oberg AL, Cunningham JM, Cerhan JR, Steer CJ, Thibodeau SN: **Gene networks and microRNAs implicated in aggressive prostate cancer.** *Cancer Res* 2009, **69:**9490-9497.

9. Sachdeva M, Mo YY: **MicroRNA-145 suppresses cell invasion and metastasis by directly targeting mucin 1.** *Cancer Res* 2010, **70:**378-387.

10. Ostenfeld MS, Bramsen JB, Lamy P, Villadsen SB, Fristrup N, Sorensen KD, Ulhoi B, Borre M, Kjems J, Dyrskjot L, Orntoft TF: **miR-145 induces caspase-dependent and -independent cell death in urothelial cancer cell lines with targeting of an expression signature present in Ta bladder tumors.** *Oncogene* 2010, **29:**1073-1084.

11. Shi B, Sepp-Lorenzino L, Prisco M, Linsley P, deAngelis T, Baserga R: **Micro RNA 145 targets the insulin receptor substrate-1 and inhibits the growth of colon cancer cells.** *J Biol Chem* 2007, **282:**32582-32590.

12. La Rocca G, Shi B, Badin M, De Angelis T, Sepp-Lorenzino L, Baserga R: **Growth inhibition by microRNAs that target the insulin receptor substrate-1.** *Cell Cycle* 2009, **8:**2255-2259.

13. Ichimi T, Enokida H, Okuno Y, Kunimoto R, Chiyomaru T, Kawamoto K, Kawahara K, Toki K, Kawakami K, Nishiyama K, et al: **Identification of novel microRNA targets based on microRNA signatures in bladder cancer.** *Int J Cancer* 2009, **125:**345-352.

14. Wang S, Bian C, Yang Z, Bo Y, Li J, Zeng L, Zhou H, Zhao RC: **miR-145 inhibits breast cancer cell growth through RTKN.** *Int J Oncol* 2009, **34:**1461-1466.

15. Martinez-Sanchez A, Dudek KA, Murphy CL: **Regulation of human chondrocyte function through direct inhibition of cartilage master regulator SOX9 by microRNA-145 (miRNA-145).** *J Biol Chem* 2012, **287:**916-924.

16. Xu T, Zhu Y, Xiong Y, Ge YY, Yun JP, Zhuang SM: **MicroRNA-195 suppresses tumorigenicity and regulates G1/S transition of human hepatocellular carcinoma cells.** *Hepatology* 2009, **50:**113-121.

17. Li D, Zhao Y, Liu C, Chen X, Qi Y, Jiang Y, Zou C, Zhang X, Liu S, Wang X, et al: **Analysis of MiR-195 and MiR-497 expression, regulation and role in breast cancer.** *Clin Cancer Res* 2011, **17:**1722-1730.

18. Findlay VJ, Turner DP, Moussa O, Watson DK: **MicroRNA-mediated inhibition of prostate-derived Ets factor messenger RNA translation affects prostate-derived Ets factor regulatory networks in human breast cancer.** *Cancer Res* 2008, **68:**8499-8506.

19. Lee Y, Yang X, Huang Y, Fan H, Zhang Q, Wu Y, Li J, Hasina R, Cheng C, Lingen MW, et al: **Network modeling identifies molecular functions targeted by miR-204 to suppress head and neck tumor metastasis.** *PLoS Comput Biol* 2010, **6:**e1000730.

20. Ernst A, Campos B, Meier J, Devens F, Liesenberg F, Wolter M, Reifenberger G, Herold-Mende C, Lichter P, Radlwimmer B: **De-repression of CTGF via the miR-17-92 cluster upon differentiation of human glioblastoma spheroid cultures.** *Oncogene* 2010, **29:**3411-3422.

21. Castellano L, Giamas G, Jacob J, Coombes RC, Lucchesi W, Thiruchelvam P, Barton G, Jiao LR, Wait R, Waxman J, et al: **The estrogen receptor-alpha-induced microRNA signature regulates itself and its transcriptional response.** *Proc Natl Acad Sci U S A* 2009, **106:**15732-15737.

22. Hayashita Y, Osada H, Tatematsu Y, Yamada H, Yanagisawa K, Tomida S, Yatabe Y, Kawahara K, Sekido Y, Takahashi T: **A polycistronic microRNA cluster, miR-17-92, is overexpressed in human lung cancers and enhances cell proliferation.** *Cancer Res* 2005, **65:**9628-9632.

23. Myatt SS, Wang J, Monteiro LJ, Christian M, Ho KK, Fusi L, Dina RE, Brosens JJ, Ghaem-Maghami S, Lam EW: **Definition of microRNAs that repress expression of the tumor suppressor gene FOXO1 in endometrial cancer.** *Cancer Res* 2010, **70:**367-377.

24. Park SM, Gaur AB, Lengyel E, Peter ME: **The miR-200 family determines the epithelial phenotype of cancer cells by targeting the E-cadherin repressors ZEB1 and ZEB2.** *Genes Dev* 2008, **22:**894-907.

25. Hyun S, Lee JH, Jin H, Nam J, Namkoong B, Lee G, Chung J, Kim VN: **Conserved MicroRNA miR-8/miR-200 and its target USH/FOG2 control growth by regulating PI3K.** *Cell* 2009, **139:**1096-1108.

26. Kitago M, Martinez SR, Nakamura T, Sim MS, Hoon DS: **Regulation of RUNX3 tumor suppressor gene expression in cutaneous melanoma.** *Clin Cancer Res* 2009, **15:**2988-2994.
